# Indents

**Published:** 1918-03

**Authors:** 


### INDENTS

**☞ Brief Articles of Technical or Scientific Value will receive notice and comment. Contributions invited.**

*Root Canal Antiseptic* Triturate equal parts of chloral hydrate and thymol in a warm mortar, adding a few drops of alcohol; a liquid mixture results which makes an ideal dressing for infected canals and putrescent pulps. It acts as a germicide and absorbent of putrefaction compounds. In opening a putrid canal use care to avoid forcing any infection through the apical foramen. This is accomplished by opening and cleaning out the pulp chamber and the openings into the roots. Place a pledget of cotton saturated with the thymol and chloral solution in the pulp chamber and cover with soft guttapercha, which should not fill the cavity in the crown. If the guttapercha filling is wiped over with a pledget of cotton saturated with chloroform it will be sealed so the medicament can't get out. Then place in the cavity a temporary cement filling to prevent injury during mastication. The dressing may be left for two or three days or longer if desirable. At the second sitting remove the dressings and with broaches cleanse the canals as thoroughly as possible, wash out with alcohol and dry with hot air. Then fill canals with sterilized cotton points saturated with the antiseptic solution and protect with cement filling as before. At the third sitting, if the canals are not sufficiently open and especially if organic material clogs the canal, use the sodium-potassium treatment for cleansing the canals. If calcic deposits interfere, use hydrochloric acid. If in doubt as to complete disinfection, renew the antiseptic treatment, or permanently fill if indications warrant. If not in doubt, but to be safe, seal in a dressing of cresol in glycerine for a few days before filling. Strict aseptic precaution should be observed in the entire treatment.—Extracted from an article by C. J. Grove, in *Journal N. D. A.*

*An Interesting Case of Focal Infection* A young lady playing in one of our local theatres, had been troubled for over a year with a general eruption of the skin, a rash covering the entire body. A chemical analysis of the make-up materials was made, thinking that perhaps impurities therein caused the eruption. For several months she was treated by skin specialists. Later had a Wassermann test made which proved negative. Finally she was advised to consult a dental surgeon. An examination of her mouth showed a severe case of gingivitis. Prophylactic treatment was given. All pockets eliminated. Her skin began to clear after a week's treatment, and in three weeks all rash had disappeared.—Dr. W. M. Wadleigh in *Pacific Gazette.*

*A Jaw Breaker* The boy's mother is said to have called Dr. R. S. Kinkel, a dentist, who found two teeth had been knocked out, the stumps remaining. On October 4th the boy's condition became so serious he was taken to the Lincoln Hospital, where it was found he was suffering from osteromiplitiso, a disease of the gums, said to be caused by blood clots forming under the teeth.—*New York Telegram.*

*Adaptation to Cavity Walls* We believe it is apparent to any thinking mind that better adaptation to cavity walls may be secured with gold foil than any other material. Indeed, gold foil is the only material with which we may use to our great advantage the elasticity of the dentin. Dentin is one of the most elastic of the tooth tissues and with gold foil we are able to spread the dentin and secure a permanent gripping of the tooth on the filling, thus securing an admirable adaptation of the gold to the cavity walls unknown in any other filling material. We thus secure a joint that is impervious to moisture, preventing the entrance of bacterial organisms, preventing tooth discoloration, and giving to the dental pulp the greatest possible protection.—Dr. J. M. Prime in Minneapolis *Journal.*

*Lower Impression* In taking lower impression, after the tray carrying the plaster has been inserted, have the patient raise the tongue as high as may be, pointing it forward; then after the tray is adjusted, drop the tongue to place. This procedure allows the thin plaster to run into every space, extending under the tongue around the edge. Do not trim this off, but preserve it sedulously, remembering that wherever the plaster runs rubber may be substituted, because it can be depended upon that plaster used as here described, very thin, will not compress or displace the tissues. The flanges so provided (by extending under the tongue) give the plate a larger bearing surface, assure the help of the tongue in holding it in place and diminish the opportunities for food to get under the denture.—Rupert F. Hall, *Dental Items of Interest.*

*Care of Children's Teeth* The subject of the care of children's teeth is one which has received insufficient attention. When I was the executive committeeman of this association upon two occasions, I tried to persuade dentists to write papers upon the subject of the care of children's teeth. It is a very difficult proposition to get members of our profession to write papers upon that subject, and Dr. Thomson, I think, has emphasized the reason that it is difficult to secure such papers—simply because we do not know how to properly treat children's teeth. And why? Is it the fault of the dental college? No! It is the fault of the dental profession. The members of the profession at large do not care to take the time to properly treat children's teeth, thereby learning the definite technic. If someone could tell us how to properly treat and fill an abscessed deciduous second molar and preserve that tooth, without preserving focal infection, and hold the space for the eruption of the bicuspid, it would certainly be a great step forward in our scientific knowledge. Not only the deciduous molars, but all of the deciduous teeth which may be pathologically involved, should be treated, filled and retained in the jaws in a healthy condition until the time for the eruption of their successors. The medical profession has had to back water in many ways. The dental profession also has been compelled to do likewise. We have had to do so in connection with pulp canal treatment. If a pulp canal is not properly filled (and I wonder what percentage were formerly properly filled), we know the pabulum remains, furnishing nourishment for micro-organisms which are constantly circulating in the patient's blood. When nonvirulent germs, relatively speaking, arrive at a focus where tissue resistance is low, such as we find at the end of an unfilled pulp canal, their virulence increases. Therefore all pulp canals of nonvital teeth must be opened and filled to their apices, and in order to do this with any degree of certainty we must check up all canal work with the X-ray.—Dr. Carl Lucas in *Dental Summary.*

*Toothache Preventable* Certainly we are not yet treating teeth as we should and it doesn't make any difference how long we talk about this subject or what is said beforehand, we always wind up facing these stolid facts: *At least ninety per cent. of toothache is preventable,* if the dentist and the patient combine their forces to prevent it. Once toothache occurs, a dental abscess may develop. But *before toothache occurs*—that is, before pulpitis—the typical *dento-alveolar abscess can not develop.*

Then why don't we start to treat teeth right, by preventing toothache? And why don't we educate the public right? So the mental attitude of the people is such that with the first stab of toothache pain the victim wails, "Oh my! There goes $50 if I am to have this tooth treated properly. And I must have it treated properly or it may ruin my health. Maybe I should have it extracted anyhow, for perhaps it can not be treated successfully, for any price. Oh, dear; Oh, dear, why did I neglect it?"

Such an attitude of mind toward toothache is the one justified by its seriousness. But neither the public nor the dentists have as yet assumed this attitude. Patients develop toothache without the slightest regret, except for the pain, and with complacent confidence that their dentist can stop the pain promptly and make the tooth "as good or better than ever."—Dr. H. R. Roper in *Summary.*

*Resolutions Sent to President Wilson by the National Dental Association*

His Excellency, President Woodrow Wilson, Executive Mansion, Washington, D. C.: Whereas, our nation is now engaged in a bitter conflict with the German empire to decide that momentous question whether democracy shall perish from earth, and Whereas, it is vitally necessary under the present existing conditions that not only individuals but also organizations state clearly and publicly their position, therefore, be it

Resolved, that we, the National Dental Association of the United States of America in convention assembled in the City of New York, do hereby extend to you our sincere sympathy and hearty endorsement by pledging our skill, our money, and, if need be, our lives.

These resolutions were unanimously adopted by a rising vote.

The secretary telegraphed these resolutions to President Woodrow Wilson, and received the following reply:

The White House,
Washington, October 23, 1917.
(Personal)

*My Dear Sir:*

The President asks me to make cordial acknowledgment of your telegram of this morning, and to tell you and everyone concerned that he deeply appreciates your patriotic assurances. With an expression of his hearty thanks, I am Sincerely yours,

J. P. TUMULTY,
*Secretary to the President.*

*A Musical Solo* Doctor—That must have been quite an interesting case you had in there? Nurse—Yes, Mrs. Lorrey just gave birth to a thirteen-pound boy. Doctor—Was it instrumental? Nurse—No, vocal.

*Is Sugar a Necessity?* The editor of the *British Dental Record,* in commenting on this question, makes the following quotations from a dentist and a physician: The dentist says that up to seventy years ago we did not have it, and now our teeth are going to pieces because of it, and our digestions will not tolerate the amount we consume. He therefore concludes that we should use less sugar or substitute other things to tickle the palate, and by so doing make up for lack of shipping tonnage. A retired consulting physician denies that sugar, at least in the quantities supposed, is not essential to the development or even the wellbeing of children, as is too commonly affirmed.

*Nerve Blocking in Fracture Treatments* The use of nerve blocking in the treatment of face and jaw wounds in war surgery should be of very great value. Complete apposition of fractured bones may be secured without pain, and so plenty of time will be had for doing careful work, and at the same time have the cooperation of the patient.—A. E. Smith, in *Dental Review.*

*To Hold Cotton Rolls in Place While Operating* Frequently a cavity may be kept dry long enough for a treatment or an amalgam filling by the use of cotton rolls without the rubber dam. The problem is to keep the rolls in place, particularly in the lower jaw, where the tongue always has a tendency to toss the rolls out of position. This may be obviated by slipping an ordinary rubber dam clamp over the tooth after the rolls are in place, allowing the beaks of the clamp to grasp a small portion of the roll between the clamp and the tooth. This will hold the roll securely and will also prevent the clamp from hurting.—*Dental Review.*

*How to Get the Best Results in Casting Watts Metal Plates with Porcelain Teeth* After the wax model has been properly invested in a casting flask allow the investing material to dry for one hour. Then place it on a low flame for forty-five minutes and on a high flame until the whole investment block is red hot. In order to prevent the checking of teeth that will occur by the different expansion or construction of Watts metal and porcelain, you have to allow your flask to cool thirty minutes. Melt your

Watts metal and pour it in the flask and allow to cool. As I have cast Watts metal for a good number of years with best results, I am positive that some fellow dentist will profit by it.—John V. Amenta, in *Review.*

*Apothesine* Carroll W. Allen, M. D., of New Orleans (*New Orleans Medical and Surgical Journal,* March, 1917), made some experiments upon himself with apothesine, in the course of which he found that the one per cent. solution produced immediate anesthesia of the skin. If injected too rapidly, a slight, negligible burning sensation, which immediately subsided, was felt at the site of the needle puncture. This sensation was not experienced when the solution was injected slowly. Complete anesthesia lasted for one hour and a quarter. A one per cent. solution of apothesine, with the addition of five drops of the 1:1000 solution of adrenalin to each ounce thereof, produced a similar result except that the pale area surrounding the wheal, produced by the injection, was more marked. The one-half of one per cent. solution of apothesine with sodium chlorid, four per cent., produced immediate anesthesia that continued thirty minutes. The addition of adrenalin solution, five drops to the ounce, prolonged the anesthesia to forty-five minutes. The one-fourth of one per cent. solution produced practically the same results. The writer concludes that in its anesthetic effect and low degree of toxicity it compares very favorably with novocain. He has performed circumcision and operations for hernia, hemorrhoids, fistula, varicocele and plastic operations with the aid of the new anesthetic, uniformly using the one-half per cent. solution, with four per cent. sodium chlorid and five drops of adrenalin chlorid solution to each ounce. The author says: "The anesthesia has been complete in all cases and has invariably lasted in excess of an hour. No immediate or late irritating effect was noted, and the wounds healed as well as after the use of any other anesthetic solution. No toxic or other unpleasant immediate or aftereffects were noted, although large quantities were purposely used to determine this point: as much as four ounces (of the solution) in one case, which represented nearly ten grains of the drug."—*Park, Davis & Co.'s Therapeutic Notes.*

*Apothesine, the New Local Anesthetic*

Aposthesine is the hydrochlorid of gamma-diethyl-amino-propyl cinnamate. It occurs as small white crystals, having a melting point of 137° C. It is readily soluble in alcohol, slightly soluble in acetone and ether, and very soluble in water. It is quite stable, and will keep indefinitely if reasonably protected from contamination. The solution in water is neutral to litmus. It is precipitated from solution by alkalis and by the ordinary alkaloidal reagents. If desirable, the solution may be sterilized in the usual manner by heating to the boiling point of water. As is the case with all anesthetics and alkaloids, the best results are insured by the use of freshly prepared solutions. It is supplied in the following forms: H. T. No. 216 (vials of 20 and 100 tablets). Each tablet contains 1¼ gr. of apothesine. One tablet dissolved in 60 minims of water—or normal saline solution—makes a two per cent. solution (approximately). H. T. No. 217 (vials of 25 and 100 tablets). Each tablet contains 3-5 gr. apothesine and 1-500 gr. adrenalin. One tablet dissolved in 60 minims of water—or normal saline solution—makes a one per cent. solution of apothesine, in adrenalin 1:30,000 (approximately). This anesthetic is a product of Parke, Davis & Co., and from reports received its toxicity is about the same as novocain, and it has the same resistance to boiling. As a local anesthetic it appears in no way inferior to novocain.—*Texas Dental Journal.*

